# The Dental Pulp, Its Pathology and Treatment

**Published:** 1876-01

**Authors:** J. B. Willmott

**Affiliations:** Toronto, Canada


### ARTICLE II.

### *The Dental Pulp, its Pathology and Treatment.*

BY J. B. WILLMOTT, D. D. S., L. D. S., TORONTO, CANADA.

Read before the American Academy of Dental Surgery of New York.

Perhaps there is no subject now prominently before the dental profession which excites more interest in the mind of the intelligent and inquiring practitioner, than the pathology of the Dental Pulp, and the most successful means of meeting pathological conditions which it presents.

In this paper it will not be so much our object to point out modes of practice, as to discuss the principles which underlie the practice, which, with slight modifications in individual cases, is now almost universally adopted by reputable practitioners, and which has on the whole, been reasonably successful.

In this field we have no expectation of being able to present anything which is not already quite familiar to most gentlemen present ; we do hope, nevertheless, to so present facts and well known principles as to awaken thought, provoke discussion, induce more thorough examination and fuller investigation, and thus indirectly be the means of adding at least one atom to our stock of professional knowledge.

For our present purpose, we may describe the Dental Pulp as a pulpy mass of reddish gray color, composed of blood vessels, nerve filaments and connective tissue, and occupying the central portion of the tooth. A large portion of the bulk of the pulp being made up of the convolutions of the capillaries and nerve filaments, it is highly vascular and exceedingly sensitive.

As would be naturally inferred from its anatomical arrangements it is very susceptible to the action of irritants. In its normal conditicn it is admirably protected from external influences by a wall of dentine, which, in its turn is surrounded by an osseous structure still more dense,—the enamel.

Within the cavity formed by these walls, the pulp is en veloped in a membraneous sac, which forms the lining membrane of the pulp chamber, and is continuous with the periosteal membrane investing the root of the tooth. The pulp itself is connected by the minute opening at the apex of the root with the nervous and circulatory systems. While the pulp and its surroundings are in a physiological condition, it performs its appointed functions with a quiet unobtrusiveness worthy of all admiration.

When, however, its privacy is violated, it promptly resents the insult by inflicting upon its unfortunate possessor the most intense agony, aptly denominated by the Scottish bard "The hell o' all diseases."

However varied may be the causes which bring about a pathological condition of this structure, the condition itself is one of inflammation, modified by the circumstances under which it exists, or it is the effect or result of inflammation. The primary causes of inflammation of the pulp and the consequent odontalgia, may be described as either external or internal irritation. Internal irritation is due to the presence of osseous deposits, either in the substance of the pulp in the form of nodules, or on the walls of the pulp chamber. The diagnosis of this pathological condition is often obscure.

When, however, in a tooth free from caries, or from caries sufficiently extensive to account for the trouble from external irritation, continuous pain located in the crown of the tooth is experienced for a considerable period, and without any soreness or percussion or other indications of periosteal trouble, and which cannot reasonably be ascribed to any reflex or sympathetic nervous action; and especially if the pain is not noticeably increased by heat or cold; we are warranted in suspecting some internal irritation. In my own practice this condition has always been associated with an unusually well developed tooth structure.

The treatment indicated by this diagnosis,—the cause being beyond our reach, would of course be to drill down and destroy the pulp, and after carefully removing it, to fill the canals and pulp chamber in the usual manner.

Of external irritants we may notice mechanical violence, thermal changes conducted by metalic plugs, and mechanical irritation, caused by foreign matter being brought into contact with the pulp in consequence of its exposure by dental caries.

In the inflammation resulting from the first of these causes, the treatment will be to allay the inflammation by soothing and cooling topical applications, and by appropriate general treatment where this may seem necessary. If, however, it cannot be terminated by resolution, it is better to devitalize the pulp than to risk periosteal complications by permitting it to end in suppuration.

In the case of thermal changes, it would seem to be wise at once to remove the filling, and after subduing the inflammation, to refill, interposing between the filling and the floor of the cavity some non-conducting substance. For this purpose the preparations of gutta percha are admirably adapted.

By far the most common cause of inflammation of this organ, is the presence of foreign matter in cavities of decay, acting either chemically or mechanically upon the exposed or nearly exposed pulp. When a case of this nature is presented for our consideration, our diagnosis must necessarily be rather inferential than direct. From the nature of the case, digital or accurate optical examination is impossible, and we are obliged to fall back on symptoms and principles. In a case of odontalgia, arising from the causes just named, from a consideration of the symptoms present and the physiological principles involved, we infer that the pulp is highly conjected, the capillaries distended and engorged, and the delicate sensitive nerve filaments pressed with considerable force against the unyielding walls of the chamber, producing the intense suffering complained of.

The exact stage at which the inflammation has arrived, is at once important and difficult to determine. We must be guided in great measure by a history of the case. If the pain has recurred frequently, and through a period of several months or even weeks, the probability is, that suppuration has taken place, resulting in a greater or less loss of substance. This condition will be more certainly indicated if the dentine surrounding the point of exposure lacks sensibility; or if passing an instrument or dry pledget of cotton over the floor of the cavity, give no increase of pain; while pressure upon a pledget of cotton saturated with saliva, increases the suffering. Notwithstanding the not infrequent declaration of eminent practitioners to the contrary, my own decided opinion is, that in such cases where a portion of pulp substance has been lost by sloughing, there is no reasonable hope of preserving the remainder alive for any considerable length of time. The experience of sixteen years practice, has only served to confiım opinion formed many years ago, that the only safe course in such cases, is the extirpation of the pulp. If, however, the inflammation has been recent, and the introduction of an instrument or dry pledget of cotton increases the pain we conclude that it is still in an acute state, without loss of substance amenable to proper treatment. This inflammation being a purely local lesion, does not usually affect the general circulation, although ın some temperaments the suffering endured does produce very marked constitutional disturbance, which must be treated on general principles. The indication as a rule, however, will be toward a local treatment. The first step will be to remove as perfectly as possible, the exciting cause of inflammation, by carefully syringing out the cavity with warm water.

The further treatment involves a consideration of the theory of inflammation. Without waiting now to discuss the various theories which have been propounded, or presuming to pronounce dogmatically in favor of any, we assume that it has been demonstrated by experiment, under the microscope, that the first effect of irritation upon contractile tissue, is contraction.

The irritation being continued, forced contraction ends in exhaustion, and as a consequence, relaxation. In the case of capillaries when relaxation takes place, the ordinary pressure of the arterial circulation distends them with blood, and in their exhausted condition they are unable unaided, to at once regain their normal condition, and an increased volume of blood flows through them. Accepting this theory, it follows that the increased determination of blood to the part, is the result of a distended condition of the capillaries, brought about by exhaustive contraction. If this assumption be correct, the obvious course of treatment in purely local inflammation, would be such topical applications as will assist in restoring the contractility and normal function of the capillaries. In general practice these conditions are found to be satisfactorily fulfilled by the stimulating astringents, such as solutions of chloride of zinc, acetate of lead, nitrate of silver, etc.

In inflammation of the dental pulp, however, better re sults have followed the use of creosote, carbolic acid, thymol, oil of cloves and similar remedies, which possess more or less of the anæsthetic property, in addition to their antiseptic and stimulating properties. The therapeutical action of these remedies is not well understood, and though extensively used for many years their use is still largely empirical. With most practitioners, probably the best reason that can be given for their use, is that observation has demonstrated that in dental practice, under favorable circumstances, they do assist in terminating inflammation by resolution.

Theoretically it would appear to be correct practice in using these agents, and especially carbolic acid, to apply them lightly and repeat the application with sufficient frequence to sustain the stimulation of the capillaries, until their normal function became restored, rather than to apply a strong solution or large quantity, cauterizing the tissue and in their depressed and abnormal condition favoring death and suppuration. In cases where the engorgement of the vessels has forced the substance of the pulp into and through the opening into the cavity of decay, a gentle pricking sufficient to cause bleeding before any topical treatment is attempted, will be found of decided advantage.

If in addition to the local symptoms presented, there is an evidently congested and clogged state of the excretory organs, the administration of an active cathartic, or sudorific, or both, will materially assist in bringing about the desired result. The pulp being restored to its physiological condition it becomes necessary to protect it from further irritation. This can only be accomplished by permanently filling the cavity of decay. The resources of the dental art have not yet furnished us with a material in all respects perfectly adapted for this purpose. What the conditions present indicate, is a material sufficiently plastic to be perfectly adapted to the wall of the cavity without pressure, and at the same time unirritating to the soft tissues, insoluble in the mouth and a bad conductor of thermal changes. Such a material our dental laboratories have not yet supplied us. The best that is available, is perhaps some of the preparations of the oxychloride of zinc.

To be effectual, these must be used in such a way that the chloride does not come in contact with the pulp. This can be secured by covering the point with a paste, made of oxide of zinc and creosote, and applying over this the oxychloride cement—covering this in turn with a permanent metallic filling. Of all the various suggestions for capping exposed pulps, this method probably offers the most encouragement in our efforts to permanently restore to comfort and usefulness a tooth in which caries have progressed until the pulp has become exposed. Time was, in the history of dental science, when the presence of an inflamed and painful pulp was, by good operators, deemed a sufficient excuse for extracting the tooth. Haunted by the ghosts of the thousands of mastications thus slaughtered, or moved with compassion for the sufferings of his patient, thus doomed to be deprived of what nature designed to be both an ornament and a necessity, some radical practitioner suggested the possibility of devitalizing the pulp, depending upon the periosteum for the nutrition of the tooth, and then filling the canal, pulp chamber and cavity of decay; and thus restoring the tooth otherwise devoted to destruction, to comfort and usefulness.

Later, the conservative practice of attempting to reduce the inflammation and preserve the exposed pulp, has been very generally adopted. With the tendency to run into extremes, which unfortunately characterizes our profession, pulps are now capped so indiscriminately, and as a consequence, with a result so unsatisfactory, that already there are many symptoms of an early return to the heroic practice of devitalization. In many cases, doubtless, failure results from want of skill or want of judgment. In most cases probably when pulps have died under an oxy-chloride filling, and when the primary conditions were favorable, the failure has resulted either from proceeding too rapidly, and capping before the normal condition of the pulp had been restored, or from applying the capping in such a way as to irritate the pulp, producing congestion, inflammation, strangulation and death.

In the great majority of cases where death occurs under these circumstances, it takes place within a comparatively few hours after the operation. It is hardly probable, as has been suggested by Dr. Dudley, of Massachusetts, that death is due to the presence of arsenic in the oxide of zinc. The rapidity with which the hydrate crystalizes, would seem to preclude the possibility of death resulting from this cause. The unfavorable results which not unfrequently follow the operation of capping, in the hands of the most skillful operators, would indicate that failure was due to the unfavorable conditions under which the operation was attempted. To be able to decide when we may safely and successfully cap, and when it is the best practice to devitalize is of the greatest importance to every operator.

In consequence of the exceptional anatomical relations of the dental pulp, it is impossible to predicate any course of treatment upcn what has been found to be successful in case of inflammation of other parts of the body. In the case of the inflammation of other soft tissues, the blood has free ingress to, and egress from the effected part.

The absorbents too are present to attempt the removal of any effusion which may take place. Under these circumstances, unless the conditions be very adverse, the tendency is to termination by resolution. In the case of the dental pulp, however, the only means of access is through a minute opening in dense unyielding osseous structure, through which not only the artery, but the nerve filament and the vein have to pass. The result of any considerable or long continued inflammation of the pulp, will produce such an increased determination of blood to the part as will so expand the artery, as to press with sufficient force upon the vein to perfectly close it, producing strangulation and consequent death of the pulp. Due consideration, will, we think, sufficiently demonstrate that nearly if not all pulps, dying from inflammation, whether caused by thermal changes, exposure, arsenic, cobalt, or from any cause whatever, die by strangulation. On this hypothesis we can account for the tendency which observation has shown to exist in acute inflammation, occurring in the pulp of a tooth, finally to terminate in death. At the same time it is proper to observe that in rare cases inflammation may recur again and again, sometimes terminating by resolution and sometimes by suppuration, and yet the tenacity of life in the pulp is so great as to survive for months and even years. In these instances it is quite probable that the opicial opening is abnormally large, and thus strangulation is avoided. On this supposition we may account for the difficulty which we sometimes experience in destroying a pulp with arsenic. In the early stages of these cases, capping would probably always be successful. Notwithstanding the peculiarly sensitive nature of the organ, and its exceptional anatomical relations, experience has demonstrated that when the exposure is but slight, the inflammation recent, the vital force strong, nutrition of the system good, constitution untainted by scrofula or syphilis, and not least in importance, when the patient attends punctually upon appointments, by proper medication the pulp may be restored to a physiological condition, and by a proper protection may be preserved in that condition. Experience teaches us on the other hand, that if the exposure is large, the inflammation long continued or of frequent recurrence, a portion of the substance of the pulp destroyed by suppuration, the circulation weak, the nutrition defective, the condition anemic, the constitution tainted, the system generally out of tone, or when any cause exists which would have a tendency to defeat our treatment, we will conserve our own interests as well as the interests of our patients, by destroying and removing the pulp, and replacing it with a proper filling.

On a consideration of the whole subject, we epitomize:

1. That the only pathological condition of the dental pulp of special interest to us, is that of inflammation.

2. That this inflammation is subject to modifications of constitution, temperament, cause, extent, duration, etc.

3. That the natural tendency is to terminate in death of the part.

4. That this tendency is mainly due to its anatomical relations, which lead to strangulation.

5. That much of the difficulty in successfully treating this inflammation, is due to this peculiarity of anatomical relation.

6. That experience teaches, that under favorable circumstances, exposed and inflamed pulps can by proper medication, be restored to a normal condition, when after capping, a suitable filling may be introduced and the tooth restored to comfort and usefulness, and be preserved in this condition.

7. That theory as well as observation teaches us, that where conditions are unfavorable, or where from any cause a reasonable doubt exists as to the success of the operation, it is both wisdom and good practice to give our reputation the benefit of the doubt and devitalize.

